# Holographic communication using programmable coding metasurface

**DOI:** 10.1515/nanoph-2023-0925

**Published:** 2024-03-08

**Authors:** Fan Zhang, Chaohui Wang, Weike Feng, Tong Liu, Zhengjie Wang, Yanzhao Wang, Mingzhao Wang, He-Xiu Xu

**Affiliations:** Air and Missile Defense College, 66488 Air Force Engineering University, Xi’an 710051, China; PLA of 93154, Jiuquan 735000, China

**Keywords:** holographic communication, spin-decoupled, programmable coding metasurface, near-field communication

## Abstract

With rapid development of holography, metasurface-based holographic communication scheme shows great potential in development of adaptive electromagnetic function. However, conventional passive metasurfaces are severely limited by poor reconfigurability, which makes it difficult to achieve wavefront manipulations in real time. Here, we propose a holographic communication strategy that on-demand target information is firstly acquired and encoded via a depth camera integrated with modified YOLOv5s target detection algorithm, then transmitted by software defined radio modules with long term evolution at 5 GHz, and finally reproduced in the form of holographic images by spin-decoupled programmable coding metasurfaces at 12 GHz after decoding through modified Gerchberg–Saxton algorithm. Experiments are conducted to demonstrate the brand-new concept of optical information conversion to electromagnetic one via above intelligent scheme. Our strategy may open a novel avenue toward applications of near-field communication based on adaptive variation of electric field patterns (i.e. holographic images).

## 1 Introduction

With rapid development of holography, holographic communication has been expected to be used in development of adaptive electromagnetic (EM) functions. It is an intelligent application scheme for information acquisition, encoding, transmission, decoding, and reproduction [1], [2]. The system realized based on above strategy can automatically generate holographic images containing specific information. Furthermore, additional techniques like beamforming can be realized by aforesaid scheme, which shows advantages of good developability and intelligence. However, related application scenarios are complex and manifest weak confidentiality. Moreover, the hardware system based on array antenna is inefficient due to their complexity, high cost, and large volume. Therefore, more flexible hardware architecture, higher-efficiency information acquisition, and more advanced theory of algorithms are urgently needed to address above issues.

Metasurfaces, as a two-dimensional version of metamaterial [3], [4], [5], afford many degrees of freedom to arbitrarily manipulate EM wavefront in terms of amplitude, phase and polarization, achieving fascinating practical applications such as diffusive scattering [6], [7], [8], [9], [10], [11], ultra-thin cloaks [12], [13], [14], [15], [16], [17], beam deflection [18], [19], [20], [21], [22], orbital angular momentum generation [23], [24], [25], [26], etc. However, reconfigurable properties cannot be realized by traditional passive metasurfaces once geometrical structures are fixed [27], [28]. More recently, active digital coding metasurfaces were proposed to dynamically control a variety of functions by integrating active components. For example, anomalous deflections and RCS reduction were realized by adopting 1-bit coding sequences (“0” and “1” states) in a single meta-atom, where “0” and “1” represent two phase states of 0° and 180°, respectively [29], [30]. Furthermore, by incorporating external control mechanisms like field-programmable gate arrays (FPGA), the digital coding metasurface concept has been extended to its programmable counterpart which allows dynamic EM manipulation with greater intelligence and intricacy [31], [32], [33], [34], [35]. Moreover, such metasurfaces with dynamically tunable properties of EM waves were also referred to reconfigurable intelligent surfaces in communication research, which serve as essential technology for wireless channel optimization [36], [37], [38].

To further improve intelligent level of metasurfaces, programmable metasurfaces have been applied to some self-adaptive applications. For example, a tracking communication system based on computer vision is proposed to improve communication quality of moving targets [39]. Moreover, a neuro-metasurface system based on supervised-evolving learning algorithms is proposed to achieve adaptive focusing [40]. These works provide creative ideas for designing programmable metasurfaces by combining advanced sensors with intelligent algorithms [41], [42], focusing on realization of adaptive functions. However, it is also important for an intelligent system to reproduce and reuse EM information. Therefore, a new strategy of metasurface-based holographic communication is highly desirable to develop adaptive EM functions, but unfortunately remains elusive so far.

Here, we propose for the first time a new strategy to achieve holographic communication by synthesizing intelligent algorithms, long term evolution (LTE) and programmable coding metasurface (PCM). Such a technique provides an intelligent solution for developing adaptive functions to convert optical information into EM one by applying accurate and real-time control. Benefiting from two independent direct current (DC) biased voltage groups supplied to two integrated PIN diodes, a spin-decoupled PCM was designed to achieve independent control of the targeted reflection field in two orthogonal circular polarization (CP) channel by a self-made microcontroller unit (MCU) development board. Moreover, depth camera and software defined radio (SDR) modules were employed to acquire and transmit target information. Therein, modified You Only Look Once version 5 small (YOLOv5s) target detection algorithm and Gerchberg–Saxton (GS) algorithm were applied for encoding and decoding. For verification, experiments were carried out, showing that our designed holographic communication system is capable of reproducing self-adaptive holographic images in real time based on particular environment. More importantly, our work offers a solid platform, expanding metasurface applications in adaptive function development.

## 2 Results and discussion

### 2.1 Application scenario and schematic architecture

**Figure 1:**
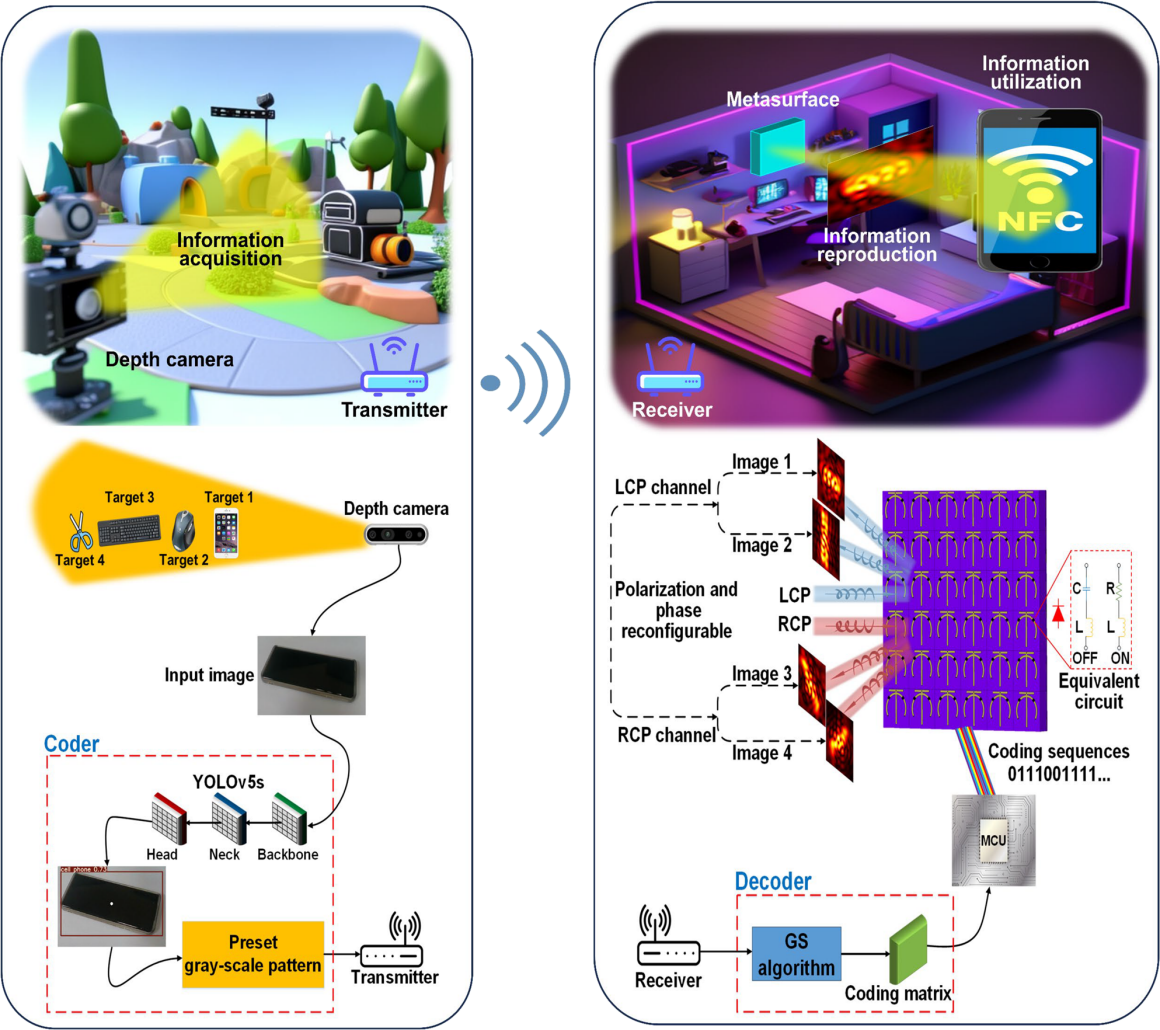
Application scenario and scheme architecture of our designed holographic communication system. Target information of selected objects is firstly automatically acquired and encoded by depth camera and modified YOLOv5s target detection algorithm, then transmitted and decoded by SDR modules and modified GS algorithm, and finally transformed into coding sequences required for spin-decoupled PCM to reproduce particular holographic image. Our proposed holographic communication scheme with polarization and phase reconfigurable feature is expected to develop adaptive EM functions, which can be further used as an NFC terminal.

Here, we conceive an application scenario of our designed holographic communication system, which is composed of spin-decoupled PCM, SDR modules, and a depth camera. Figure 1 illustrates schematic diagram of corresponding application scenario. Target information in the form of images is firstly acquired by a depth camera (Intel RealSense D435i) under complicated environment, then scaled to [608, 608, 3] before encoding, and finally fed to YOLOv5s target detection algorithm for prediction of classification results. The preliminary encoding procedure takes around 0.07 s to identify targets. Therein, each image is firstly collected at a rate of 30 FPS (frames per second), and then processed in the neural network within about 0.03 s. Furthermore, aforementioned real-time procedure makes it possible to recognize moving objects well. To further complete encoding, we map classification results to preset gray-scale image patterns by optimizing the algorithm to not only detect targets but also extract desired encoded information. Here, we use cell phone, mouse, keyboard and scissors as target examples in experiments. With the help of SDR modules ADALM-PLUTO and LTE toolboxes in MATLAB commercial software, baseband reference measurement channel waveform carrying information will be modulated to hardware memory for continuous transmission in free space.

After the received information is demodulated, modified GS algorithm is used to decode it into a coding matrix rather than original data for information transformation with good confidentiality. Then, decoded results are transformed into coding sequences for applying to spin-decoupled PCM which can realize phase modulation of EM wave in left-handed circular polarization (LCP) and right-handed circular polarization (RCP) channels by controlling left and right PIN diode states at different DC voltages. As can be seen from the equivalent circuits of PIN diodes at two states shown in middle part of right panel of Figure 1, the values of *C*, *L,* and *R* are 0.03 pF, 0.03 nH, and 7.8 Ω, respectively. Finally, holographic images are adaptively reproduced in particular CP channels according to variational target information. As a result, optical information conversion into EM one can be realized progressively.

Considering subsequent application of EM information, our proposed strategy for holographic communication is expected to develop adaptive EM functions, which can be further used as a near-field communication (NFC) terminal to establish point-to-point communication with other NFC terminals by continuously reproducing versatile EM fields in near field sensing region, realizing exciting applications including access control, mobile identity identification, and anti-counterfeiting, etc.

### 2.2 Encoding and decoding

**Figure 2:**
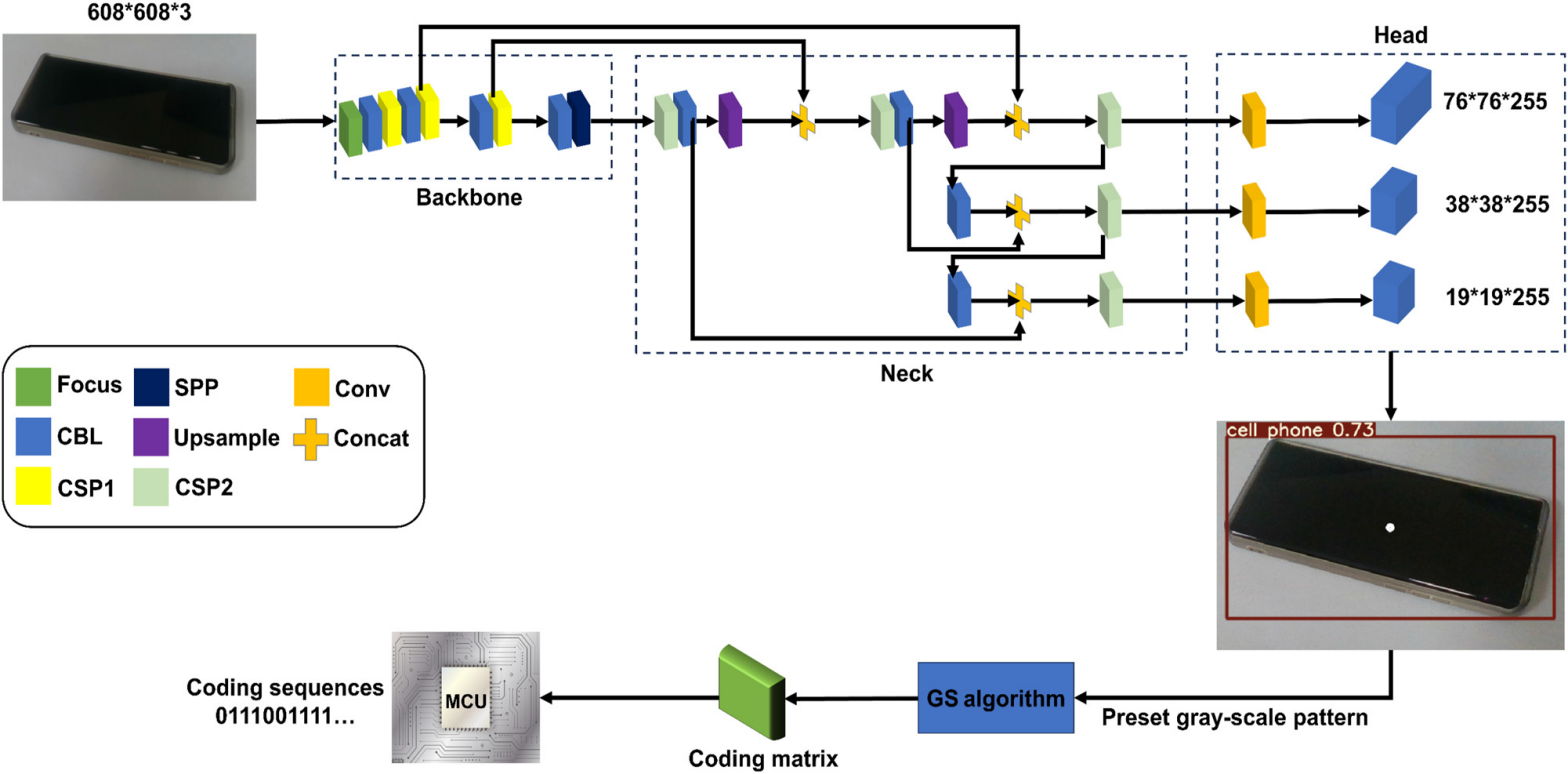
Structure diagrams of modified YOLOv5s and GS algorithm. Acquired target information is firstly fed into modified YOLOv5s target detection algorithm for classification result prediction and gray-scale image pattern mapping, then fed into modified GS algorithm for coding matrix evaluation, and finally transformed into coding sequences which are sent to MCU development board for real-time reproducing of automatic images.

As mentioned previously, depth cameras adopted in our work can realize acquisition of three-dimensional coordinates and achieve versatile functions. Here, it was used to encode acquired target information for simple verification. More specifically, sampled images were processed to obtain accurate classification results and mapped gray-scale image patterns by using modified YOLOv5s target detection algorithm for its strong network with appropriate depth and width. Therein, new CSP-Darknet53 network with strong feature extraction ability is introduced to realize high-precision detection with high speed, as shown in Figure 2. By loading COCO pre-trained weight and adopting NVIDIA GeForce RTX 4080 GPU, the time used to train the network was reduced to 5 h. Moreover, adaptive image scaling and mosaic data enhancement were used for preprocessing to adaptively scale images to [608, 608, 3] and expand data sets from 300 to 2000 with low GPU storage occupation, solving the issue of low average accuracy induced by different image sizes and uneven distribution of small targets in large data sets. In this regard, detection efficiency and integrity of image data can be improved significantly.

To obtain feature maps with different dimensions [76, 76, 255], [38, 38, 255], and [19, 19, 255] for ultimate classification results, preprocessed images are firstly put into backbone (New CSP-Darknet53) network for feature extraction, then fed into neck (SPPF, New CSP-PAN) and head network for operation and prediction. Finally, a mapping relationship between classification results and preset gray-scale image patterns should be implemented to optimize YOLOv5s algorithm, more details are presented in Note S1 of Supporting Information.

Here, modified GS algorithm is applied for decoding based on Rayleigh–Sommerfeld diffraction theory to analyze required phase profiles, which can be expressed as [43]:

(1) $$U_{h}\left(x_{h},y_{h}\right)=\frac{1}{i\lambda }\underset{s_{0}}{\iint }U_{0}\left(x_{0},y_{0}\right)\cos\left\langle n,r\right\rangle \frac{\exp\left(ikr\right)}{r}\text{d}S_{0}$$

where *U*_*h*_(*x*_*h*_, *y*_*h*_) and *U*_0_(*x*_0_, *y*_0_) stand for E-field distribution on holographic images plane and imaging plane, respectively. *λ* and *k* denote operating wavelength and wave number in free space, respectively. $r=\sqrt{\left[{\left(x_{h}-x_{0}\right)}^{2}+{\left(y_{h}-y_{0}\right)}^{2}+F^{2}\right]}$ is distance between (*x*_*h*_, *y*_*h*_, 0) on meta-holographic images plane and (*x*_0_, *y*_0_, *F*) on imaging plane, and $\cos\left\langle n,r\right\rangle =\frac{F}{r}$ . *S*_0_ denotes imaging region.

To retrieve ultimate coding matrix, it is essential to discretize continuous Equation (1) and adopt binarization and iterative method. The detailed process is presented in Note S2 of Supporting Information. Finally, evaluated coding matrixes are transformed into form of sequences, and then sent to MCU development board for automatic reproduction of holographic images.

### 2.3 Information transmission scheme

**Figure 3:**
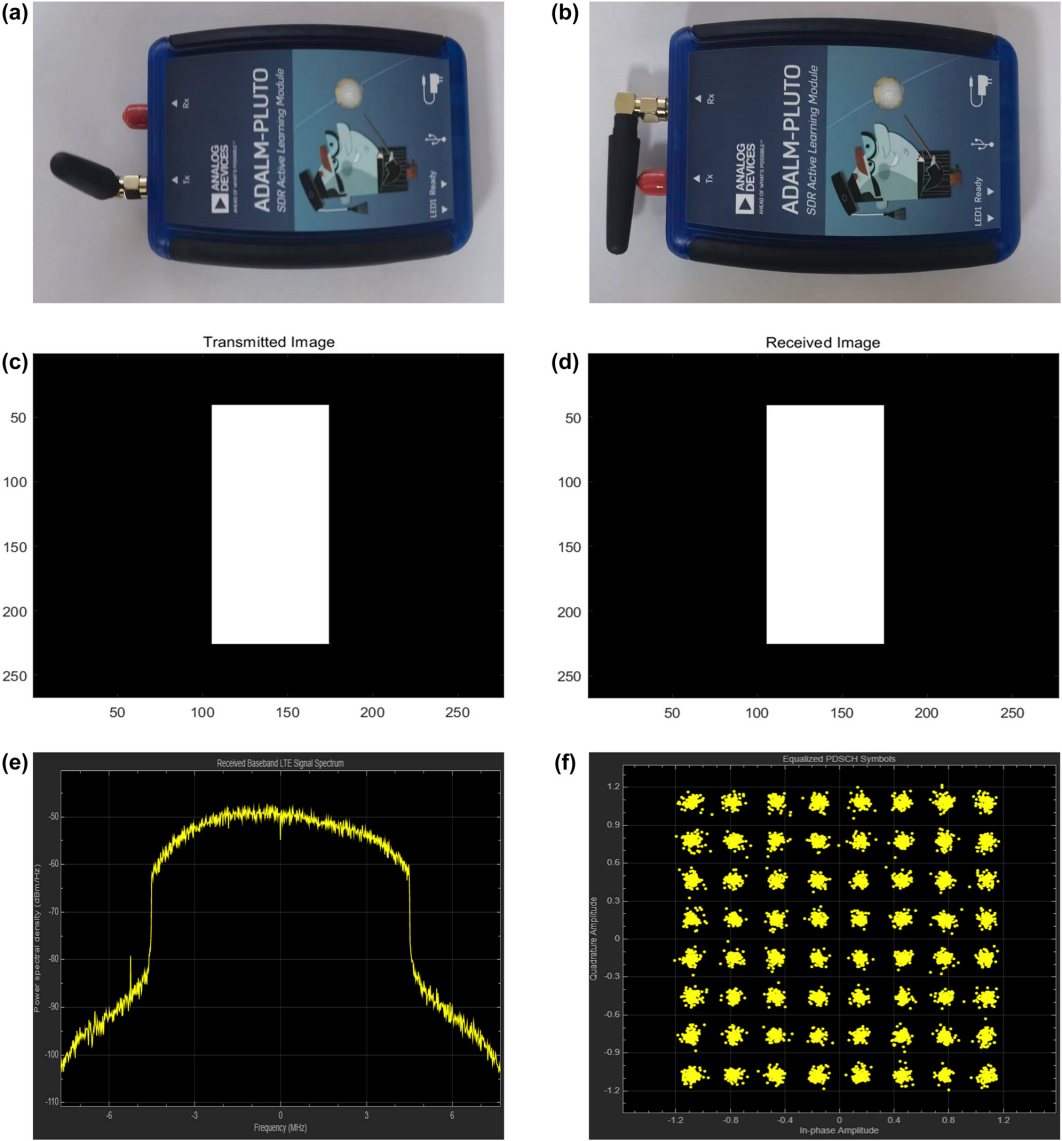
SDR modules and related measured results. (a–b) SDR modules used for transmitting and receiving information. (c) Transmitted gray-scale image pattern. (d) Received gray-scale image pattern. (e) Received baseband LTE signal spectrum. (f) Constellation diagram of equalized physical downlink shared channel symbols.

The system based on multi-terminal collaboration can extend spatial dimension and enrich substantially application scenarios, fulfilling increasingly complex application requirements. Therefore, a stable scheme adopting two SDR modules ADALM-PLUTO is proposed here to achieve reliable information transmission between two terminals, see the hardware shown in Figure 3a and b. For experimental verification, cell phone targets were used as an experimental sample. Furthermore, LTE toolbox in MATLAB commercial software was applied to modulate preset gray-scale image patterns into transmitted radio frames, and then demodulate corresponding received data to original image patterns. Moreover, detailed procedure is illustrated below for great understanding.

By converting into binary data stream, gray-scale image patterns were packed into transport blocks of a downlink shared channel to generate baseband LTE signals, which were applied to an SDR module for continuous transmission at a center frequency of 5 GHz. After capturing abundant frames by another SDR module, offsets of captured signals were determined and corrected firstly. And then, processed information was synchronized to the start of LTE frames for demodulation to get resource grids. Finally, a channel estimation has been performed. Therein, downlink shared channel was analyzed to obtain raw data which were then recombined to form ultimate image patterns.

To reflect performance of our proposed information transmission scheme, transmitted and received image patterns are shown in Figure 3c and d. Besides, power spectral density of captured signal and constellation diagram of equalized physical downlink shared channel symbols are shown in Figure 3e and f. Therein, the spectrum shows that transmitted bandwidth of signal is approximately 9 MHz. Moreover, the constellation diagram shows that noise is randomly distributed around the ideal value in forms of cloud, indicating that the signal is slightly disturbed by a white noise. In addition, root mean square error of vector magnitude and bit error rate are 2.431 % and 0.001 % in measurement, demonstrating that the designed scheme is functioning well and image patterns have retained comparatively complete information throughout the transmission process.

### 2.4 Meta-atom design and EM property

**Figure 4:**
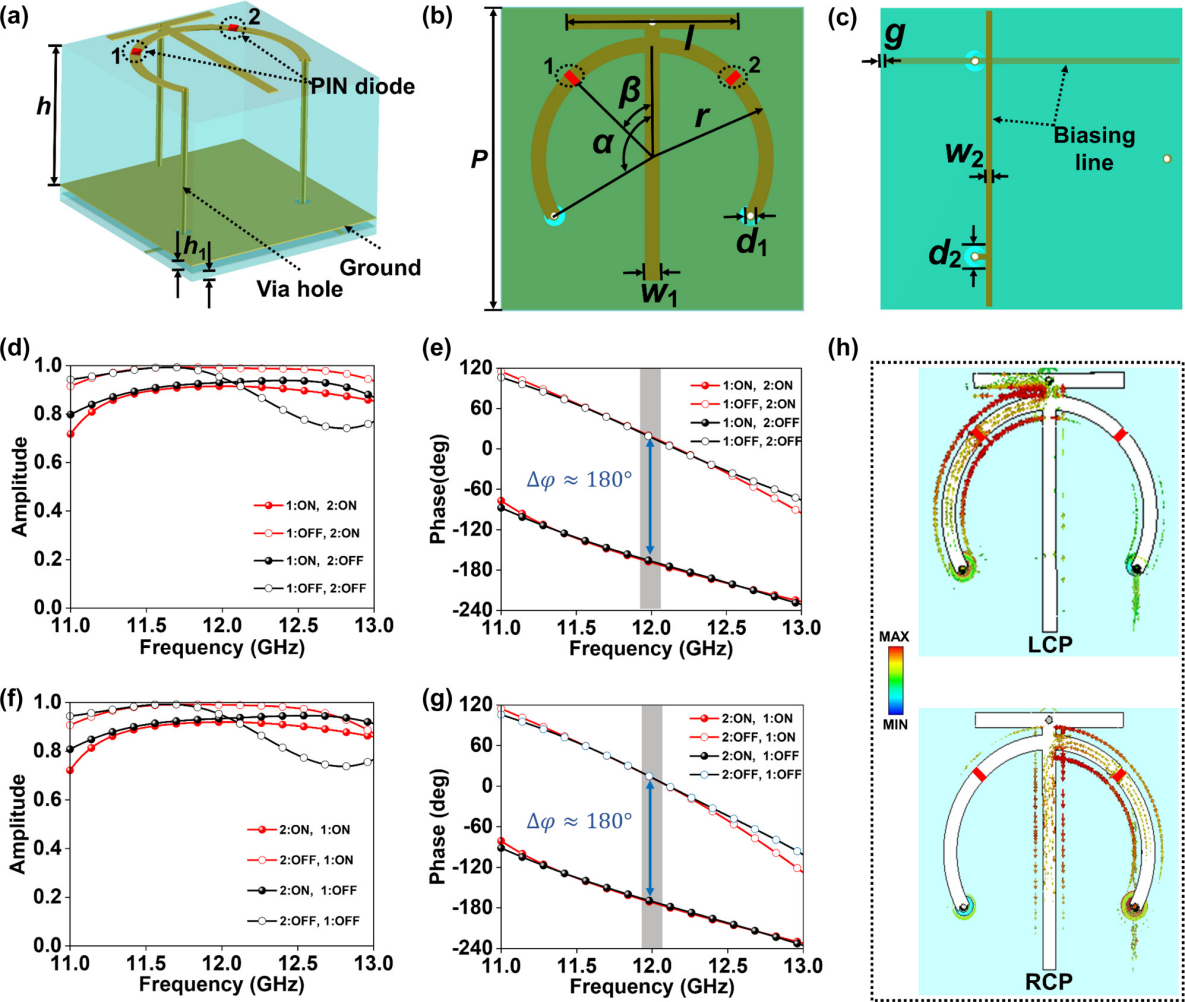
The schematic view of our designed meta-atom and its simulated co-polarized performances. (a) Perspective, (b) top, and (c) bottom view of the programmable meta-atom. Simulated co-polarized (d–f) reflection amplitudes and (e–g) reflection phases responses of the proposed meta-atom with four different PIN diode combination states under orthogonal CP wave incidence. (h) Simulated surface current distribution of the spin-decoupled meta-atom under orthogonal CP wave incidence.

To encrypt aforementioned information in different polarization channels for holographic communication applications, polarization-multiplexing metasurfaces should be used here. However, there are no related technology being used to achieve independent control of EM wave functions using AA (Aharonov–Anandan) phase used in our meta-atom design under two orthogonal linear polarization channels. Therefore, a CP metasurface was used in our work rather than a linear polarization counterpart. Here, AA phase is defined as the geometric phase accumulated in rotation path i.e. phase offsets in CP channels can be varied by changing length of meta-atom along rotation direction [44]. To construct a PCM working under two orthogonal CP incident waves based on AA phase, a triple-layer reflective element is designed with spin-decoupled phase control and high reflection coefficient, which provides stable reflection responses in orthogonal CP channels. As shown in Figure 4a, the designed programmable meta-atom is composed of four layers with diverse metallic patterns. Three F4B spacers (with a relative permittivity of 2.65 + 0.001*i*) separate the layers, thickness of which are *h* = 3 mm and *h*_1_ = 0.2 mm, respectively. In addition, the top layer is an umbrella-type resonator, the middle layer is metallic ground and the bottom two are mutually orthogonal biasing lines. Then two PIN diodes (denoted as “1” and “2”) are loaded in two arcs of umbrella type, respectively. To simplify control circuit, two PIN diodes share same ground by a metallic via hole to connect negative poles of each diode, while positive poles are connected to different biasing lines by two metallic via holes without any connection with ground. As shown in Figure 4b, two PIN diodes are loaded in the position and vertical-symmetrical one of *β* = 45°, respectively, which is beneficial for generating 180° phase difference under two orthogonal CP channels between diodes states “ON” and “OFF”. In Figure 4c, two biasing lines with a gap of *g* = 0.1 mm are orthogonally printed in upper and lower layers, ensuring independent phase control of each element by applying DC bias voltages. Other parameters are given as follows: *P* = 10 mm, *r* = 4 mm, *l* = 3.4 mm, *α* = 135°, *d*_1_ = 0.7 mm, *w*_1_ = 1 mm, *w*_2_ = 0.2 mm, and *d*_2_ = 0.9 mm.

To investigate reflection properties of our designed programmable meta-atom, we perform finite-difference-time-domain (FDTD) calculations in CST Microwave Studio. In Figure 4d and e, we give simulated reflection amplitude and phase under LCP wave excitation. Here, the overall working states of the meta-atom are denoted as “(diode 1, diode 2)” with each PIN diode 1, 2 ∈ [ON, OFF], i.e. only two possible states for each diode. It is obvious that reflection amplitude maintains over 0.9 and phase difference is 180° between states “ON” and “OFF” of PIN diode 1 at 12 GHz. Moreover, the calculated results indicate that diode 2 states are immune to LCP wave incidence, which guarantees independent phase control in LCP channel. Next, we explore EM response of meta-atom under RCP wave incidence when states of PIN diode 2 are switched under “ON” and “OFF”, see Figure 4f and g. Simulated results show that the programmable meta-atoms achieve a high reflection rate of more than 0.9. Meanwhile, phase difference of 180° is generated between states “ON” and “OFF” of diode 2 at 12 GHz. Similarly, there is little influence on EM properties when switching the states of diode 1 to “ON” or “OFF” state in RCP channel. Therefore, our proposed meta-atom indeed achieves 1-bit spin-decoupled phase control of CP wave. To further verify above independent control of two orthogonal CP channels, surface currents are simulated, as depicted in Figure 4h. Results show that the induced surface current flows along the left and right arc under LCP and RCP wave incidence, respectively.

### 2.5 Holographic communication system and experiments

In the following, we establish a system to experimentally validate the performance of our proposed holographic communication scheme. Therein, the spin-decoupled PCM fabricated based on printed circuit board (PCB) technology was composed of 21 × 21 meta-atoms, where 2 × 21 × 21 PIN diodes were embedded on metal surface, more details are shown in Note S3 of Supporting Information. Moreover, the DC bias voltages of each PIN diode were provided by a self-made MCU development board (detailed information is given in Note S4 of Supporting Information).

**Figure 5:**
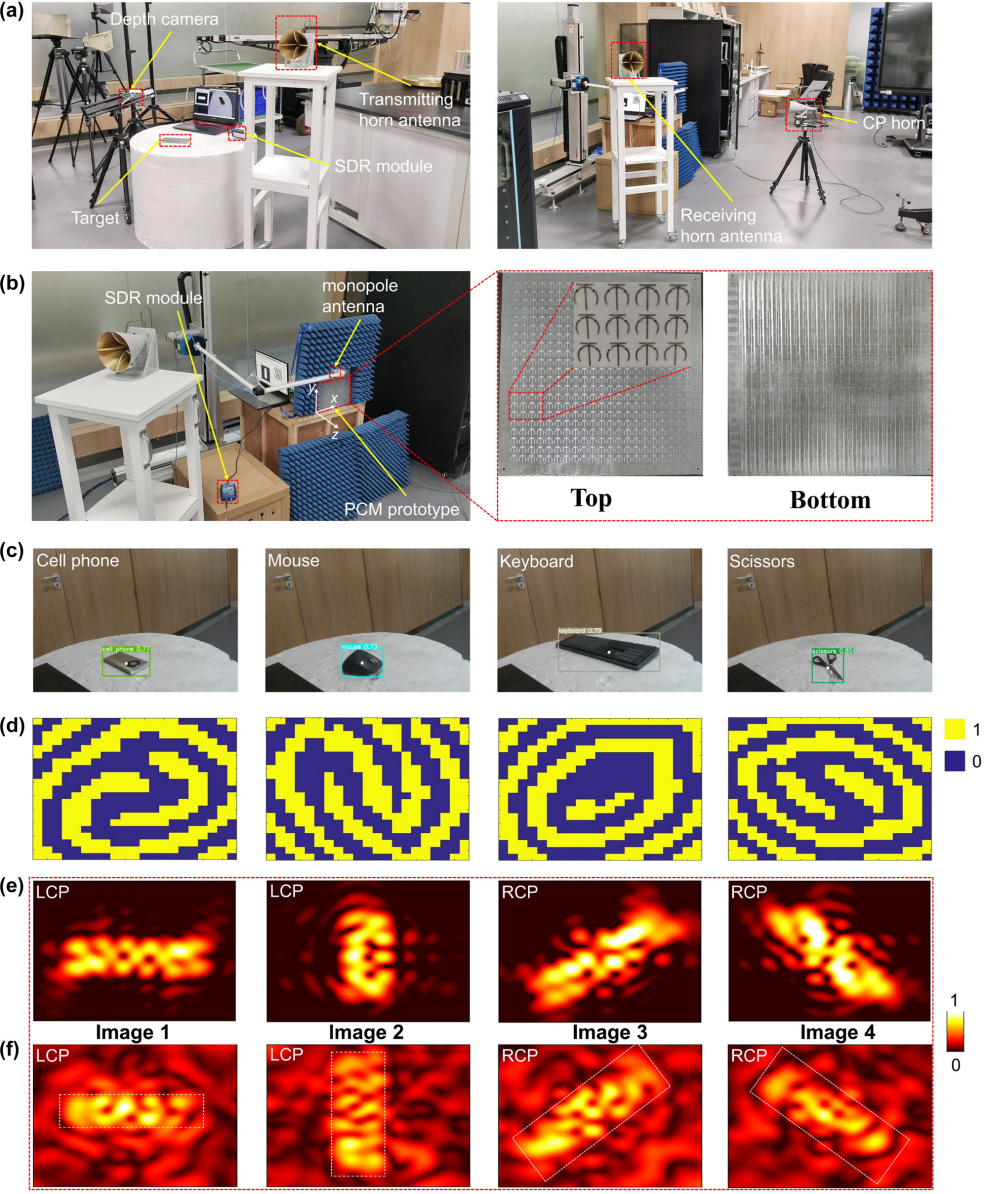
Simulated and measured results of our designed holographic communication system and experiment setups. (a) Experimental setups at transmitter (left panel) and receiver (right panel) end. (b) Additional experimental setups at receiver end (left panel) and details on the PCM prototype (right panel). (c) Predicted classification results by modified YOLOv5s target detection algorithm. (d) Retrieved coding matrix by modified GS algorithm. (e) Simulated and (f) measured near-field electric field distributions of holographic images. Each column corresponds to the same target.

Experimental setups and PCM prototype details of the holographic communication system are presented in Figure 5a and b. Therein, three-dimensional coordinate system is provided and forward direction is defined as the orientation along −*z*. At the transmitter end, variational target information was firstly fed into modified YOLOv5s target detection algorithm for encoding to predict classification results and obtain mapped gray-scale image patterns, the prediction outcome during detection process is presented in Figure 5c. After encoding, LTE-based SDR modules connected with high-power horn antennas were employed for reliable directional transmission. At the receiver end, received information was decoded into coding matrix based on modified GS algorithm, visualized results of measurement were presented in Figure 5d. Moreover, corresponding simulated near-field electric field distributions of holographic images as shown in Figure 5e. And then, coding sequences transformed from matrix form were applied to PCM in the form of DC bias voltage by MCU development board, the detailed control process of DC bias voltage is presented in Note S5 of Supporting Information. Here, PCM was excited by a switchable CP horn from forward incidence, and intensity distribution of reflected wave was captured by a 6 mm-long monopole antenna placed at 120 mm away from *x* to *y* plane. Furthermore, the incident wave in free space was deducted from the total fields to obtain targeted reflection field. In all measurements, a CP horn and a monopole antenna were connected to two ports of an AV3672B vector network analyzer, respectively. To obtain local *Ex* and *Ey* field patterns, a monopole antenna was connected with a two-dimensional electronic step motor which could move automatically in a maximum area of 0.21 m × 0.21 m with a step resolution of 4.2 mm. Then, LCP and RCP components could be calculated by incorporating both measured information according to $E_{\text{LCP}}=\frac{1}{\sqrt{2}}\left(E_{x}-iE_{y}\right)$ , $E_{\text{RCP}}=\frac{1}{\sqrt{2}}\left(E_{x}+iE_{y}\right)$ . Ultimately, measured results of holographic images corresponding to four classification results are illustrated in Figure 5f, demonstrating that our proposed strategy can continuously reproduce versatile electric field patterns (i.e. holographic images) in near field sensing regions according to variational target information, and detailed demo video is supplemented in Supplementary Movie 1. As shown in Figure 5e and f, image 1/2 and image 3/4 were reproduced in LCP channel and RCP one, respectively. With the spin-decoupled holography, EM information is encrypted in different CP channels for heightened security. It is evident that measured near-field electric field distributions of holographic images are in good agreement with simulated ones, and all performances are consistent well with our design, which fully proved that our holographic communication system is with high efficiency and can be used to develop adaptive function. Finally, quantitative analysis was carried out to evaluate the quality of images [45]. An overall efficiency of ∼50 % (defined as the ratio of the field intensity of holographic images to total incident energy) is achieved in our design. It is lower than simulated ones (above ∼80 %) due to the machining error of PCM prototype, the non-ideal plane wave illumination and the imperfect PIN diodes, etc. We also achieved a signal-to-noise ratio of ∼3 in PCM sample (defined as the ratio of peak intensity in images to the standard deviation of background noise). To improve above performance in the designed system, the 1-bit phase modulations of our PCM can be readily extended to multiple bits. In addition, it is also available to use complex amplitude modulation by extending current phase-only design to amplitude-phase design for accurate EM wave control.

## 3 Conclusions

In this work, we propose a new strategy of holographic communication with self-adaptive capacity to reproduce versatile holographic images in real time based on particular environment. With depth cameras and SDR modules, variational target information is captured and transformed into coding matrix required for PCM based on modified YOLOv5s and GS algorithms. Moreover, a spin-decoupled PCM is designed to control targeted reflection field in two orthogonal CP channels by a self-made MCU development board. As a prototype, a holographic communication system is constructed to verify our design and experimental verifications have been carried out to prove that our proposed strategy paves a new way for continuous reproduction of versatile electric field patterns (i.e. holographic images), which can be further extended in application of NFC. In future, three-dimensional coordinates of targets acquired by depth cameras will be utilized to achieve broader application scenarios with abundant information. In addition, more advanced NFC terminals using our designed system can be designed for establishing point-to-point communication.

## Supporting Information

Mapping relationship between classification results and preset gray-scale image patterns; Decoding using modified GS algorithm; Additional information for programmable coding metasurface; Design of self-made MCU development board; Detailed control process of DC bias voltage.

## Supplementary Material

Supplementary Material Details
