# Supplementary material for: Holographic communication using programmable coding metasurface
Source: Nanophotonics. 2024 Mar 8;13(8):1509–19. doi: 10.1515/nanoph-2023-0925 (PMC11635963; doi:10.1515/nanoph-2023-0925)
Supplement: Supplementary file 1 — Supplementary Material Details [file j_nanoph-2023-0925_suppl_001.docx]

**Supporting Information**

**Holographic communication using programmable coding metasurface**

Fan Zhang^1^, Chaohui Wang^1†^, Weike Feng*^1^, Tong Liu^1^, Zhengjie Wang^1^, Yanzhao Wang^1^, Mingzhao Wang^2^, He-Xiu Xu*^1^

^1^ *Air and Missile Defense College, Air Force Engineering University, Xi’an 710051, China*

^2^ *PLA of 93154*

## *Corresponding Author

E-mail: hxxuellen@gmail.com (He-Xiu Xu)

E-mail: fengweike007@163.com (Weike Feng)

† This author contributed equally to this work

**KEYWORDS:** Holographic communication, spin-decoupled, programmable coding metasurface, near-field communication

1. **Mapping relationship between classification results and preset gray-scale image patterns**
2. **Decoding using modified GS algorithm**
3. **Additional information for programmable coding metasurface**
4. **Design of self-made MCU** **development board**
5. **Detailed control process of DC bias voltage**

## Mapping relationship between classification results and preset gray-scale image patterns

Complex holographic images are hard to be reproduced well due to 1-bit phase modulation of our designed programmable meta-atom. Therefore, we construct a mapping relationship between classification results and preset gray-scale image patterns, to realize transformation of target information into EM one. In our work, cell phone, mouse, keyboard and scissors were used as examples in experiments, corresponding to rectangular gray-scale image patterns with four different rotation angles, as shown in **Figure S1.**


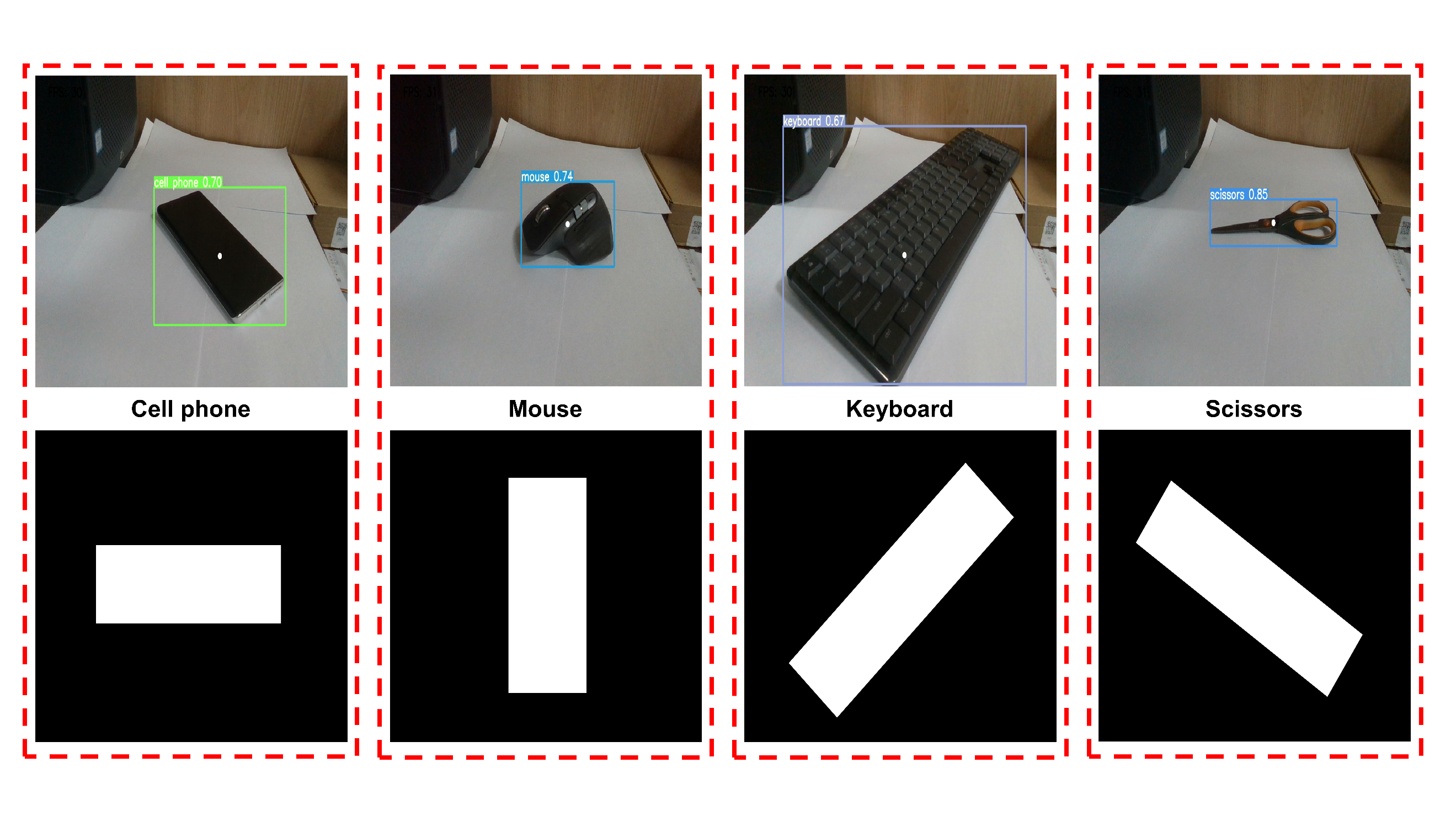


**Figure S1** Mapping relationship between classification results and preset gray-scale image patterns.

## Decoding using GS algorithm

Overall, coding matrix for arbitrary holographic images can be determined based on modified GS algorithm by performing program codes in mathematical software, see **Figure S2.** Here, A and B are intermediate results, *U*_h_ is a phase profile of required holographic images, *U*_0_ is a preset gray-scale image pattern to be projected, $\varphi_{c}$ is a coding matrix after phase binarization using a fixed threshold method. Inverse diffraction integral is the same as diffraction integral, except for that exponent in the integral is replaced by its complex conjugate. This is because in inverse case, it deals with the backward propagation of wave.

The reason to perform rigorous evaluation instead of conventional fast Fourier transform is that the experimental configuration in this case does not satisfy paraxial approximation condition, i.e., $z^{2}>>l^{2}$, where $z=r\cdot cos\left\langle n,r \right\rangle$ and *l* is lateral dimension of holographic images. Evaluation of diffraction solution using Fourier transform is the result of Fresnel approximation, which in turn is the result of paraxial approximation.





**Figure S2** Detailed decoding of retrieving coding matrix according to arbitrary holographic images based on modified GS algorithm.

## Additional information for programmable coding metasurface

With successful design of the 1-bit programmable meta-atoms, spin-decoupled PCM with dynamical holography can be realized. Here, fabricated PCM is composed of 21 × 21 1-bit programmable meta-atoms and its configuration is schematically shown in **Figure S3a,** where 21 × 21 meta-atoms are homogeneously placed on a dielectric surface. Moreover, PIN diodes are loaded in each meta-atom, **see Figure S3b.** As shown in **Figure S3c,** to ensure independent phase control of each element, control circuit of each PIN diode is properly designed on the bottom two layers to supply independent DC bias voltage groups by metallic via holes placed on four frames.


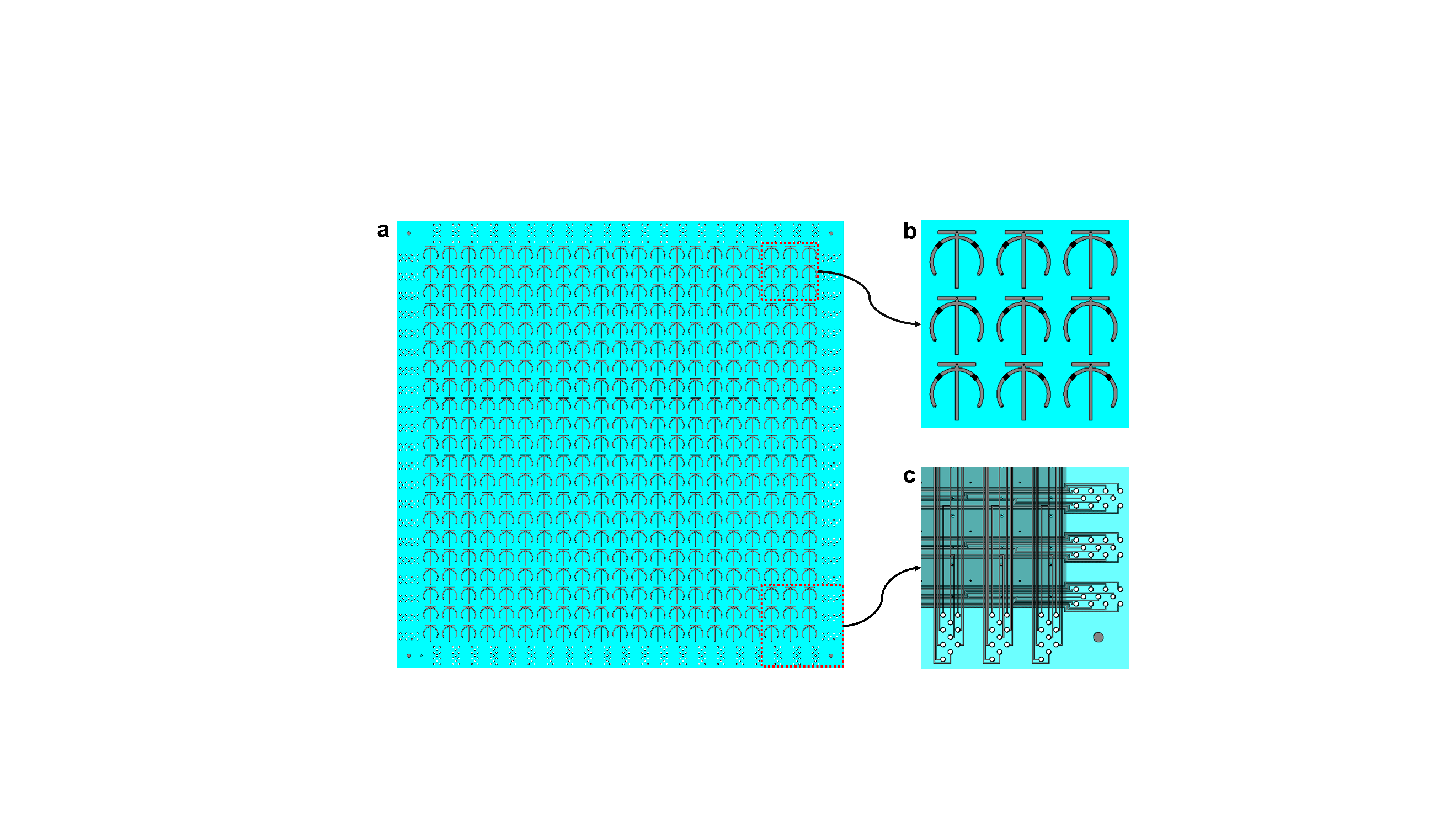


**Figure S3** Final configuration of the 21×21 PCM with (a) global top, (b) local top and (c) local bottom view.

## Design of self-made MCU development board

To realize more flexible control of EM waves, intelligent controllers with powerful performance are required to be designed. As a chip-level computer, MCU is becoming more and more popular due to their simple system structure, high reliability, robust process function, low power consumption and good environmental adaptability. Therefore, it is widely used in microelectronics applications such as cell phone, robot control and so on. The basic structure of MCU includes central process unit (CPU), storage units, input and output (IO) ports, timer and bus, as shown in **Figure S4a.** Therein, CPU is consists of an arithmetic logic unit (ALU) and a control unit (CU). Moreover, it is also a brain of MCU for reading, decoding and executing instructions to perform operations of arithmetic, logical and data transfer. In terms of storage units, a program memory are used to store instructions while data memory is used to store temporary data. Here, program memory is read-only memory (ROM) and data memory is random access memory (RAM). Furthermore, timers are used for function generation, modulation of pulse width and clock control. To achieve connection of CPU and other peripherals, bus system is used here.

The working principle of MCU is the process of executing pre-stored programs. Therefore, problems to be solved must be firstly compiled into a series of instructions, then identified and executed by a separate function. Therein, instructions is trasformated to a program for automatic perform of specific task. Finally, these programs need to be prestored in memory with storage capacity for subsequent execution. Based on above theory, we designed a 900-IO MCU development board prototype, as shown in **Figure S4b.**

The designed PCM is composed of a large number of programmable meta-atoms with integrated diodes. Therefore, it is necessary to design a MCU development board of multi-IO control to adaptively realize EM functions. Here, we use multiple MCU of STM32H743 series to expand IO ports. Moreover, a central MCU is used to invoke IO resources of other sub-MCU in parallel communication, as shown in **Figure S5.** In addition, we designed a periphery including power supply, serial communication and sensor modules, as shown in **Figure S6.** To provide reliable power supply for corresponding functional modules, LM111MPX chip is used for voltage reduction and current limiting, which supplies required energy power to different modules. Meanwhile, a large-size screen is designed for visual control of all outputs. Moreover, various sensor modules and key modules are designed to enrich the peripheral functions of MCU development board.


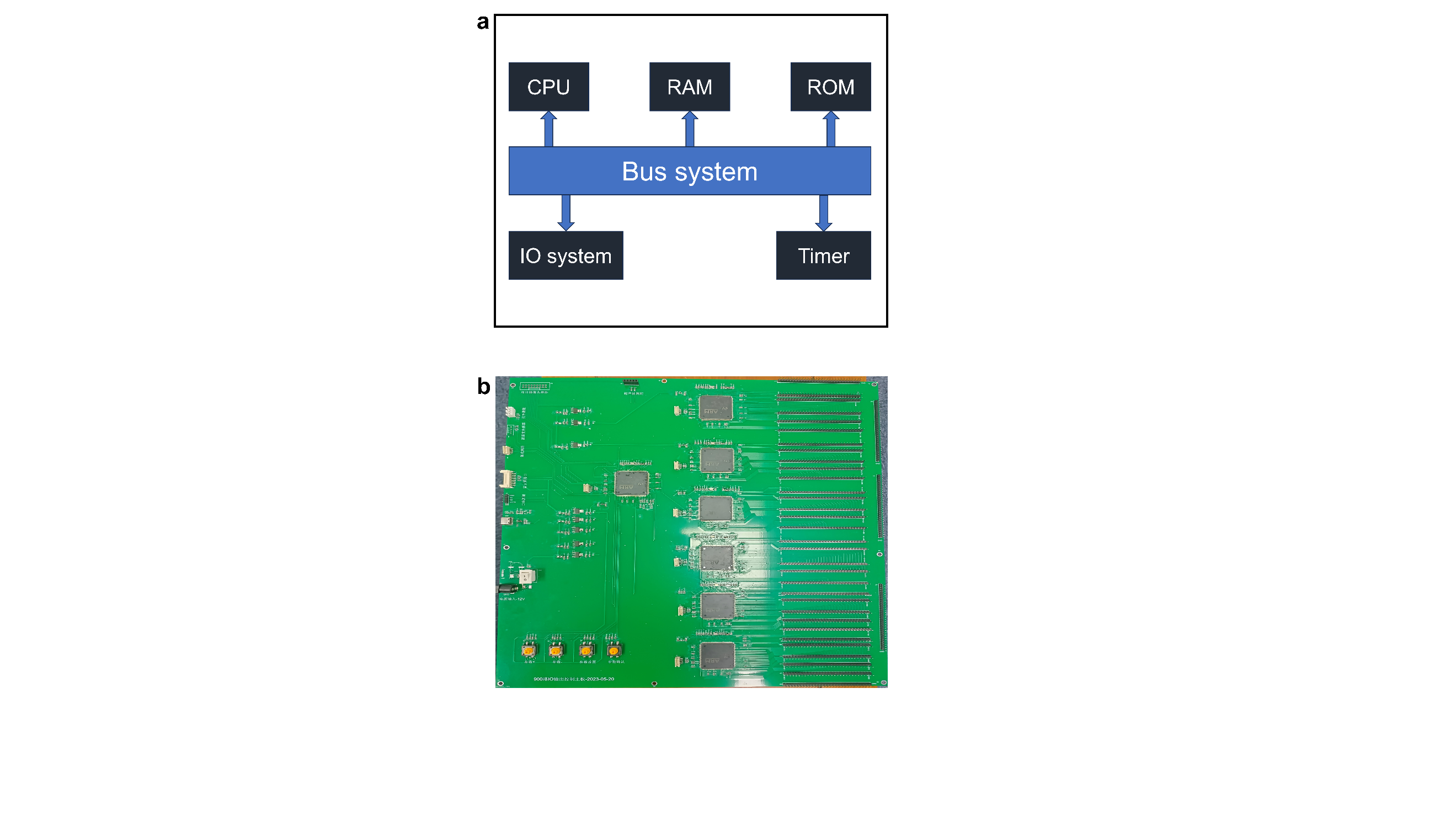


**Figure S4** (a) Basic structure and (b) Prototype of self-made MCU development board.





**Figure S5** Part 1 of self-made MCU development board circuit schematic diagram.





**Figure S6** Part 2 of self-made MCU development board circuit schematic diagram.

## Detailed control process of DC bias voltage

To achieve flexible programmability of MCU development board to achieve control of DC bias voltage process, we need to build a connection between MCU and computer. Here, we adopt USB-to-serial port module based on CH340 to realize serial communication between MCU development board and computer. The module presented in **Figure S7a** has advantages of high speed and less consumption of pin resources. Meanwhile, a large-size screen is used to achieve visualization of IO port control, as shown in **Figure S7b.** During measurement, the screen display the real-time coding matrix corresponding to versatile holographic images, as shown in **Figure S7c-f.**


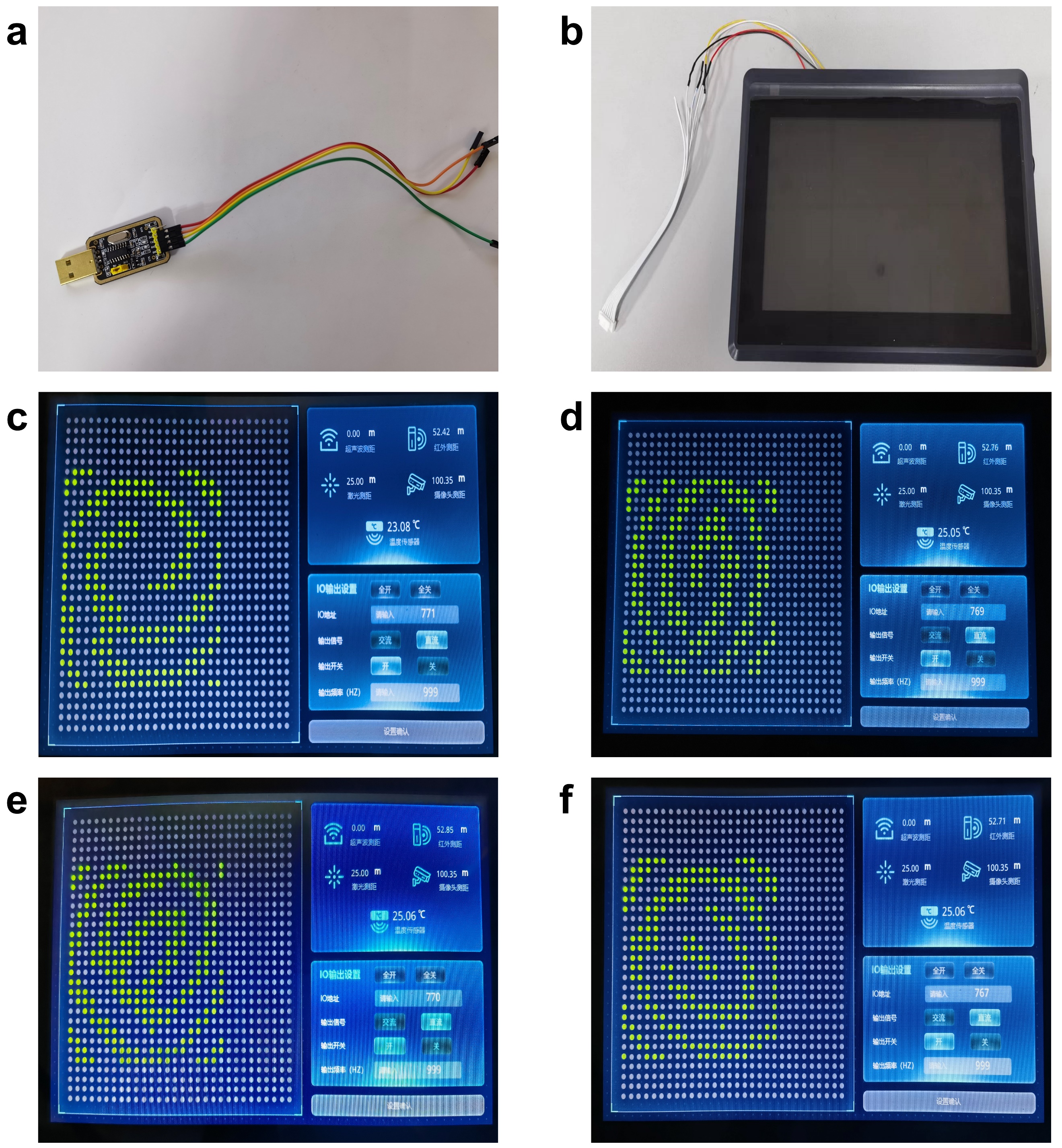


**Figure S7** (a) Hardware required for IO port control, (b) MCU development board and (c-f) related measurement results of coding matrix corresponding to versatile holographic images.
